# Resveratrol for Cancer Radio‐Sensitization: Ready for Prime Time or Future Perspective

**DOI:** 10.1002/fsn3.71579

**Published:** 2026-05-06

**Authors:** Maryam Fallah, Ali Soleimani, Ali Nouri, Melika Fallah, Alireza Haghighi, Ahmad Reza Dehpour, Abolfazl Zendehdel, Soraiya Ebrahimpour‐Koujan

**Affiliations:** ^1^ Department of Clinical Nutrition, School of Nutritional Sciences and Dietetics Tehran University of Medical Sciences Tehran Iran; ^2^ Department of Clinical Nutrition and Dietetics, Faculty of Nutrition and Food Technology, National Nutrition and Food Technology Research Institute Shahid Beheshti University of Medical Sciences Tehran Iran; ^3^ Research Center for Biochemistry and Nutrition in Metabolic Diseases, Institute for Basic Sciences Kashan University of Medical Sciences Kashan Iran; ^4^ Department of Medicine Brigham and Women's Hospital and Harvard Medical School Boston Massachusetts USA; ^5^ Broad Institute of MIT and Harvard Cambridge Massachusetts USA; ^6^ Department of Pharmacology, School of Medicine Tehran University of Medical Sciences Tehran Iran; ^7^ Geriatric Department, Ziayian Hospital Tehran University of Medical Sciences Tehran Iran; ^8^ Cancer Research Center, Cancer Institute Tehran University of Medical Sciences Tehran Iran

**Keywords:** anticancer, apoptosis, autophagy, radio‐sensitization, radiotherapy, resveratrol

## Abstract

Resveratrol, a flavonoid with antioxidant and anti‐inflammatory properties, shows therapeutic potential in metabolic, neurological, cardiovascular, viral, allergic, and inflammatory diseases. Preclinical studies demonstrate its mechanisms, including modulation of oxidative stress and inflammatory pathways, while clinical trials suggest benefits in anti‐cancer and cardioprotective applications. Resveratrol modulates inflammatory and survival pathways by inhibiting ROS production and suppressing NF‐κB and JAK/STAT signaling through the prevention of key phosphorylation and nuclear translocation events. Concurrently, it affects metabolic signaling via AMPK‐dependent inhibition of mTOR, leading to altered transcriptional activity. However, inconsistent clinical outcomes and potential side effects, such as gastrointestinal discomfort (e.g., nausea, diarrhea) and drug interactions at high doses, highlight significant limitations. This study reviews clinical data on the effects of resveratrol on disease‐specific biomarkers, emphasizing its potential as an adjunct in cancer radiotherapy. The variability in efficacy and safety underscores the need for well‐designed clinical trials to validate therapeutic benefits and assess risks. To address these gaps, we propose a comprehensive framework for future research, incorporating standardized methodologies, optimized dosing regimens, and rigorous safety evaluations. This framework aims to clarify resveratrol's efficacy, address its safety concerns, and facilitate its clinical application. By providing a structured roadmap for future investigations, this study seeks to balance the understanding of resveratrol's therapeutic potential with its limitations, paving the way for evidence‐based clinical use.

## Graphical abstract

Resveratrol exerts antioxidant and anti‐inflammatory effects by reducing reactive oxygen species and suppressing NF‐κB and JAK/STAT signaling pathways. It modulates metabolic signaling through AMPK‐dependent inhibition of mTOR, influencing cell survival and inflammatory responses. Despite promising preclinical and clinical evidence—particularly as an adjunct in cancer radiotherapy—variable efficacy and dose‐related adverse effects highlight the need for standardized clinical trials and optimized dosing strategies.

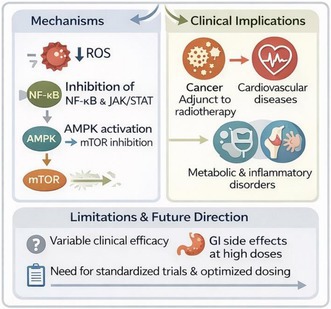

## 1 Background

The therapeutic effects of plant‐derived components in various diseases have created a new field in nutrition science. Among these plants, researchers pay great attention to flavonoid compounds because of their different therapeutic properties. The difference between flavonoid compounds is due to their variety and quantity. Resveratrol is a phytoalexin from the stilbenoid class found in many plant species (Carter et al. 2014; Khattar, Khan, Zaidi, et al. 2022; Chimento et al. 2016). Studies have investigated the therapeutic effects of resveratrol in numerous diseases, including a wide range of cancers, cardiovascular diseases, diabetes, inflammatory diseases, viral diseases, etc. (Carter et al. 2014; Chen et al. 2015; Frazzi and Tigano 2014; Oyenihi et al. 2016; Bonnefont‐Rousselot 2016; Yang et al. 2015; Campagna and Rivas 2010). Studies have shown the therapeutic mechanisms of resveratrol, which reduce inflammation and cancer progression. Based on the published articles, the anti‐inflammatory effects of resveratrol have been proven in the stages of initiation, promotion, and progression of cancer (Aggarwal and Shishodia 2005; Athar et al. 2007). Also, resveratrol shows its anti‐inflammatory effects by modulating the pro‐inflammatory mediators (Das and Das 2007a). This study will focus on the nutritional aspects (such as structure, bioavailability, metabolism, nutritional properties, and safety of the supplement) and the therapeutic aspect of resveratrol on the modulation of inflammation and oxidative stress in various stages of cancer treatment based on cellular action mechanisms. Plant‐derived secondary metabolites span a continuum from predominantly radioprotective to mainly radiosensitizing compounds, depending on their redox properties, molecular targets, and cellular context (Li et al. 2016; Zhang et al. 2023).

Many phytochemicals, including polyphenols and polysaccharides from medicinal plants, act as radioprotectors of normal tissues by scavenging radiation‐induced reactive oxygen (ROS) and nitrogen species (NOS), reinforcing endogenous antioxidant systems, supporting DNA‐damage repair pathways, and modulating inflammatory and hematopoietic responses (Li et al. 2016; Zhang et al. 2023).

In parallel, several plant‐derived agents such as curcumin, genistein, and quercetin can behave as context‐dependent radiosensitizers in tumor cells by amplifying radiation‐induced DNA damage, enforcing cell‐cycle arrest, suppressing pro‐survival signaling networks, and interfering with DNA‐repair machinery, while still providing some degree of radioprotection in non‐malignant tissues at appropriate doses and schedules (Zhang et al. 2023).

Within this spectrum, resveratrol emerges as a functional polyphenol: on the one hand, it can mitigate radiation toxicity in normal organs through antioxidant, anti‐inflammatory and pro‐repair effects, and on the other hand, in various cancer models it increases oxidative stress, attenuates DNA‐damage response signaling and promotes apoptosis, autophagy or mitotic catastrophe, resulting in overall enhancement of tumor radiosensitivity when combined with radiotherapy (Carter et al. 2014; Khattar, Khan, Zaidi, Darvishikolour, et al. 2022). Cancer radiotherapy comprises three major categories: external beam radiotherapy (EBRT), brachytherapy, and systemic radionuclide therapy, defined by the location of the radiation source relative to the patient's body (Koka et al. 2022).

EBRT has evolved from simple two‐dimensional techniques to highly conformal three‐dimensional conformal radiotherapy (3DCRT), intensity‐modulated radiotherapy (IMRT), volumetric modulated arc therapy (VMAT), image‐guided radiotherapy (IGRT), stereotactic body radiotherapy (SBRT), and particle therapy with protons or heavy ions, enabling precise dose delivery to tumors while sparing surrounding normal tissues in a wide range of malignancies, including head and neck, lung, breast, prostate, and central nervous system tumors (Koka et al. 2022; Chen et al. 2023; Jaffray 2012).

At the biophysical level, ionizing radiation exerts its cytotoxic effect predominantly through the induction of DNA double‐strand breaks, created via both direct ionization of DNA and indirect damage mediated by radiolysis of water and generation of reactive oxygen species; high‐LET particles such as neutrons and carbon ions produce dense, complex lesions with higher relative biological effectiveness than conventional low‐LET photons (Koka et al. 2022; Chen et al. 2023).

These lesions activate DNA damage response pathways and programmed cell‐death (apoptosis, mitotic catastrophe, senescence) and modulate the tumor vasculature, microenvironment, and anti‐tumor immune responses, which together shape the therapeutic index and provide multiple molecular targets for pharmacological radiosensitisation strategies (Koka et al. 2022; Chen et al. 2023).

## 2 Nutritional Aspects

### 2.1 Structure and Bioavailability

Resveratrol (3,5,4′‐trihydroxystilbene) is a natural polyphenol with beneficial biological activities to improve human health (King et al. 2006). This polyphenol compound is produced by some spermatophytes, such as peanuts and grapes, in response to injury (Frémont 2000). The polyphenolic structure exhibits antioxidant activity and reduces the apoptosis induced by oxidants and low‐density lipoprotein (LDL) (King et al. 2006). The biological activities of trans‐resveratrol (trans‐3,4′,5‐trihydroxystilbene; t‐RES) and its analogs depend on its structure, which includes: double bond, position and number of hydroxyl groups, stereoisomery, and intramolecular hydrogen bonding (Ovesna and Horvathova‐Kozics 2005; Afaq and Katiyar 2011). According to the double bond and bearing ortho‐diphenoxyl or para‐diphenoxyl functionalities of t‐RES can be modified (Ovesná et al. 2006). Having a 4′‐hydroxy group possesses a more effective anticancer effect than other analogues (Ovesna and Horvathova‐Kozics 2005). About 70% of oral resveratrol is rapidly absorbed and metabolized, and its half‐life is 9–10 h in the body (Gadag et al. 2022). Oral absorption of resveratrol is facilitated by transepithelial diffusion and is about 75% (Walle 2011). Increasing the dose and frequency of dietary intake did not show a significant effect on its absorption (Walle 2011). From a nutritional and supplemental perspective, resveratrol has been administered in human studies across a broad dose range, typically from 10s to several 100s of milligrams per day, with single doses up to 5 g generally reported as well tolerated in the short term (Berman et al. 2017).

Despite its relatively low oral bioavailability caused by rapid first‐pass metabolism and extensive sulfate and glucuronide conjugation, these data indicate a favorable safety profile for resveratrol as a dietary supplement (Walle 2011).

This safety background, together with preliminary clinical experience in oncology, provides a rationale for exploring resveratrol as an adjuvant in cancer treatment, including its potential role as a radiosensitizing agent in clinical settings (Carter et al. 2014; Berman et al. 2017).

### 2.2 Resveratrol Metabolism

Each substance has a specific place to be metabolized in the human body. The main sites of resveratrol metabolism are the liver and intestine (Walle 2011). Resveratrol metabolism has been processed by intestinal microflora and hepatic detoxification (Walle et al. 2004). Resveratrol has low solubility in water and must be bound to protein, such as lipoproteins, hemoglobin, and albumin, to be transferred (Belguendouz et al. 1997; Jannin et al. 2004). The cytochrome P450 enzyme CYP1B1 converts resveratrol to piceatannol, an active anticancer compound (Potter et al. 2002; Szekeres et al. 2010). Resveratrol has a very low bioavailability due to its rapid metabolism and production of sulfate and glucuronide metabolites (Wenzel and Somoza 2005). Gene expression of sulfotransferase 1A1 (SULT1A1) enzyme, which converts resveratrol to 3‐O‐sulfates, reduces the anticancer activity of resveratrol in human breast cancer cells (Szekeres et al. 2010; Murias et al. 2008). However, piceatannol, which is a more active metabolite than the sulfated form, does not reduce the anticancer activity (Szekeres et al. 2010; Murias et al. 2008).

### 2.3 Nutritional Properties

Similar to other flavonoids, resveratrol has anti‐oxidative properties, and free phenolic groups have been considered necessary for its effects (Zhang and Tsao 2016; Li et al. 2016). Based on experimental efforts, potential biological effects of dietary resveratrol include neuroprotective, anti‐inflammatory, anti‐diabetic, and hypolipidemic‐hypoglycemic properties, as well as chemopreventive effects against neoplasia and cardiovascular disease (Carter et al. 2014; Chen et al. 2015; Frazzi and Tigano 2014; Oyenihi et al. 2016; Bonnefont‐Rousselot 2016; Yang et al. 2015; Campagna and Rivas 2010). Resveratrol acts as a signaling pathway modulator, which affects tumorigenic and/or carcinogenic pathways. It is involved in many transcription factors' gene expression. The anticancer effects of resveratrol are: suppression of pro‐inflammatory signaling pathways that cause cancer progression, inhibition of carcinogen activation and induction of carcinogen detoxification, growth arrest, and induction of apoptosis (Whitlock and Baek 2012; Sun et al. 2008; Aluyen et al. 2012; Dadgostar et al. 2021; Shafabakhsh et al. 2023).

## 3 Clinical Application

Beyond oncology, resveratrol has shown beneficial effects in metabolic disorders such as obesity and diabetes, where it improves insulin sensitivity, modulates glucose homeostasis and attenuates cardiometabolic risk factors through AMPK‐ and SIRT1‐dependent pathways (Öztürk et al. 2017; Szkudelski and Szkudelska 2011).

These systemic actions are tightly linked to a broad anti‐inflammatory profile, including suppression of NF‐κB signaling, down‐regulation of pro‐inflammatory cytokines and modulation of eicosanoid synthesis (Das and Das 2007a; de la Lastra and Villegas 2005).

In parallel, resveratrol exhibits antiviral and immunomodulatory activities against several human pathogens, largely by reducing oxidative stress, inhibiting viral gene expression and fine‐tuning innate and adaptive immune responses (Campagna and Rivas 2010; Yang et al. 2015).

Collectively, these non‐oncological data suggest that resveratrol can reshape the inflammatory, metabolic and immune function at the whole‐organism level, factors that are known to influence tumor biology, normal tissue tolerance and ultimately the response to ionizing radiation in clinical radiotherapy (Szkudelski and Szkudelska 2011; Das and Das 2007a).

### 3.1 Anti‐Inflammatory Effects

**FIGURE 1:**
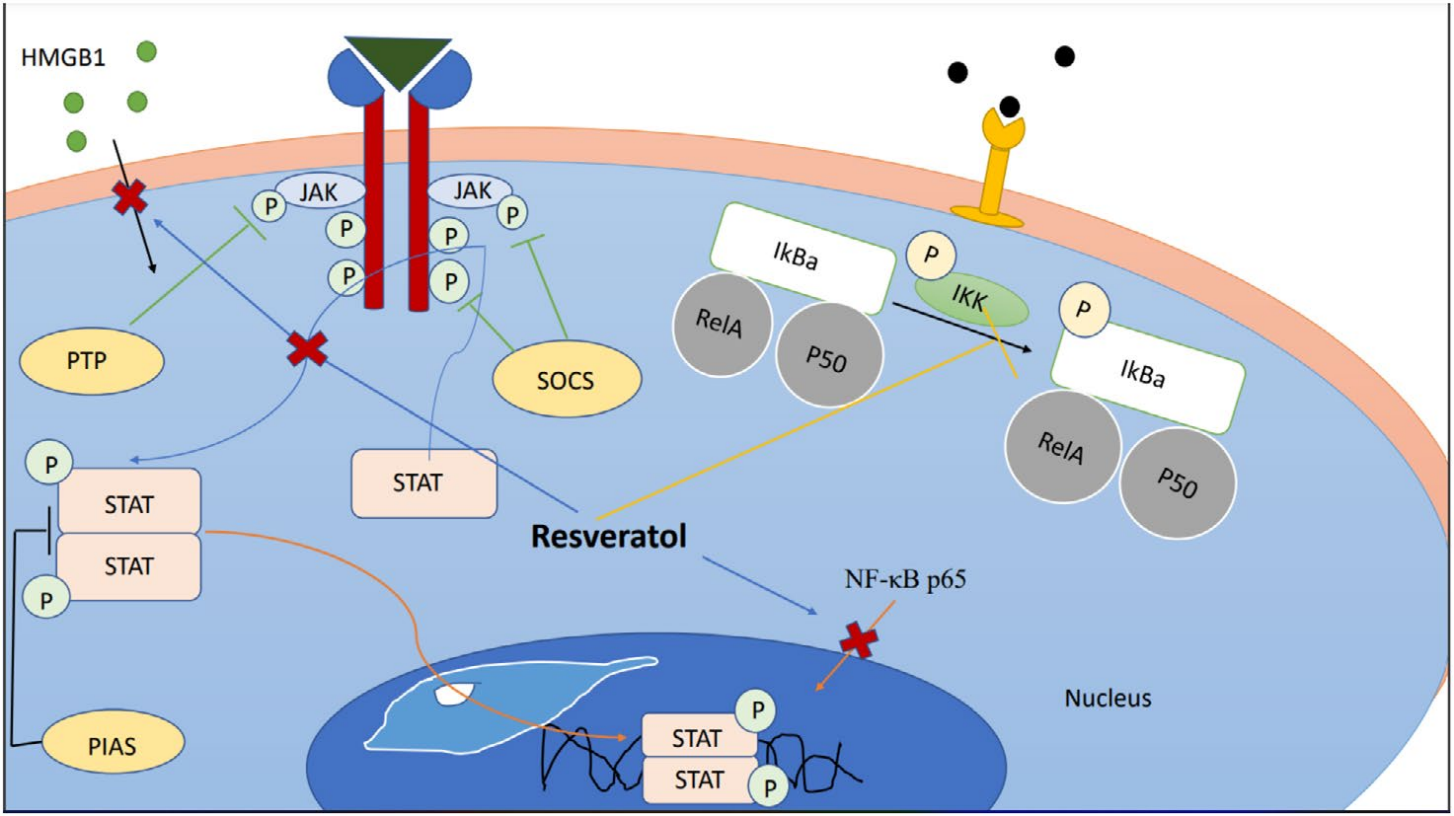
Resveratrol prevents IKBa phosphorylation. It also prevents NF‐κB p65 entry into the nucleus. It prevents HMGB1 entry into the cell. Inhibiting the phosphorylation of STAT prevents phosphorylated STAT from entering the cell. HMGB, high mobility group box 1; JAK, Janus kinase; *P*, phosphorus.

Resveratrol significantly reduces MCP‐1 secretion and MCP‐1 promoter activity caused by TNF‐α (Zhu et al. 2008). Also, resveratrol decreases the transcription of adiponectin, adipocytokines, and IL‐6 in a dose‐dependent manner (Zhu et al. 2008). Resveratrol supplementation reduces the activity of the NF‐kappa B pathway caused by TNF‐α (Uchida et al. 2005). Also, Resveratrol can stimulate Sirt1 protein acetylation (Lakshminarasimhan et al. 2013). Sirt1 can inhibit the transcription of RelA/p65 subunit of NF‐kappa B by deacetylating RelA/p65 at lysine 310 (Borra et al. 2005; Yeung et al. 2004). The effect of resveratrol on Sirt1 can reduce MCP‐1 gene expression caused by TNF‐α (Zhu et al. 2008). The findings show that increasing the activity of Sirt1 can be a possible mechanism of resveratrol in the expression of MCP‐1 (Zhu et al. 2008). Also, resveratrol decreases the transcription of the iNOS gene expression and IL‐6 mRNA (Ma et al. 2015). Another anti‐inflammatory effect of resveratrol is related to the inhibition of HMGB1 transfer to the cytoplasm (Ma et al. 2015). Also, resveratrol inhibits the translocation of the p65 subunit of NF‐κB from cytosol to the nucleus and decreases the phosphorylation of IκBα in a dose‐dependent manner (Ma et al. 2015). Resveratrol causes an anti‐inflammatory effect on HMGB1 and IL‐6 via suppression of the JAK/STAT pathway Figure 1 (Ma et al. 2015). Also, this flavonoid can reduce the phosphorylation of STAT1 and STAT3 (Ma et al. 2015). Resveratrol inhibits the transcription of NF‐kB and activator protein‐1 (AP‐1) gene expression, which, as a result, reduces the expression of TNF‐α‐mediated matrix metallopeptidase (MMP)‐9 (Zhu et al. 2008; Jarrahian et al. 2000). Another possible effect of inhibiting NF‐kB by resveratrol can be the reduction of cell adhesion (Pendurthi and Rao 2002). Resveratrol reduces NF‐kB binding activity and inhibits TNF‐α‐induced MCP‐1 expression in adipocytes (Kremeyer et al. 2010). Resveratrol inhibits the transcription and activity of cyclooxygenase‐2 (COX‐2), as well as reducing COX enzymes (Martinez and Moreno 2000; Jang and Pezzuto 1998; Kanduja et al. 2004). It can exert its anti‐inflammatory effect by activating peroxisome proliferator‐activated receptor (PPAR) (Inoue et al. 2003). Resveratrol is capable of inhibiting COX‐2, which can reduce the synthesis of PGE2. This polyphenol can prevent neutrophil infiltration and colon damage and leading to a decrease in PGD2 (Alarcon De La Lastra and Villegas 2005). Resveratrol reduces COX‐2 activity and its activation by PMA by inhibiting protein kinase C (PKC) and activator protein‐1 (AP‐1) (Subbaramaiah et al. 1998). It can also increase apoptosis and decrease cell proliferation (Kuo et al. 2002). Resveratrol decreased neutrophil adhesion and ICAM‐1 and VCAM‐1 gene expression by human umbilical vein endothelial cells stimulated with TNF‐α (Alarcon De La Lastra and Villegas 2005). In addition to reducing the adhesion of neutrophils, resveratrol inhibits the adhesion of monocytoid U937 cells to endothelial cells stimulated with LPS (Pendurthi and Rao 2002). It can also reduce the adhesion of neutrophils to EA.hy.926 cells, which is stimulated by TNF‐α (Alarcon De La Lastra and Villegas 2005). Resveratrol interferes with the pro‐inflammatory signaling of thrombin, which can reduce the activity of neutrophils by inhibiting the protease‐activated receptor (PAR), reducing the release of adenosine nucleotides from activated platelets, and also inhibiting P2 receptor signaling by interfering with c‐Jun NH2‐terminal kinase (JNK) and mitogen (mitogen‐activated protein kinase [MAPK]; Alarcon De La Lastra and Villegas 2005).

As a result, resveratrol polyphenol can be effective on neutrophil–platelet interaction and the release of free radicals by inhibiting the release of inflammatory adenosine derivatives and reducing the activity of platelets by reducing the response to thrombin (Kaser et al. 2004).

**FIGURE 2:**
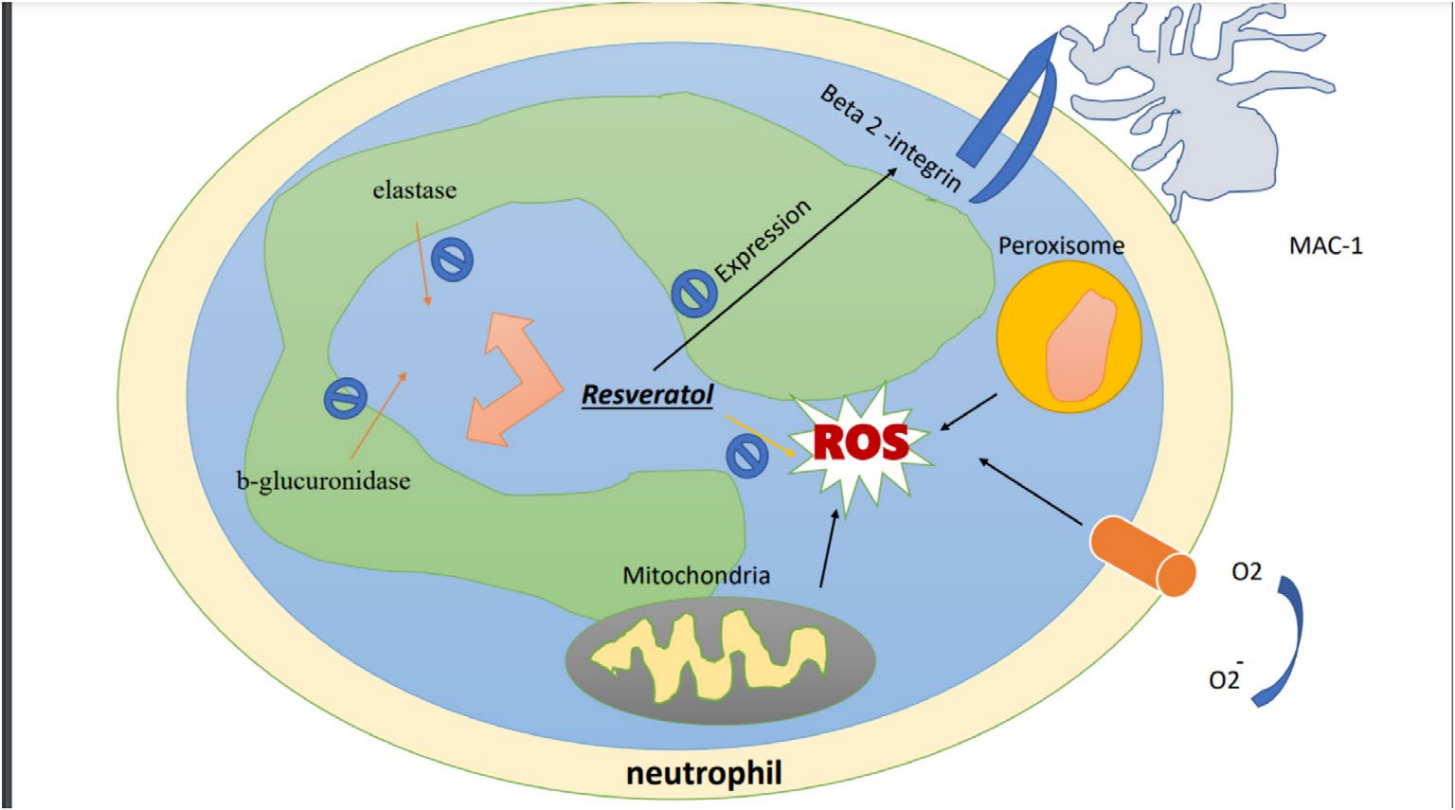
Resveratrol inhibits β‐glucuronidase and elastase. It inhibits the expression of Beta 2‐integrin. It inhibits ROS, which is made by peroxisome and mitochondrial activities. MAC, macrophage; ROS, reactive oxygen species.

Resveratrol inhibits the release of elastase and β‐glucuronidase from neutrophil granules, the production of reactive oxygen metabolites (ROM), and also reduces the expression of the β2‐integrin MAC‐1 on PMN surface Figure 2 (Rotondo et al. 1998). Resveratrol reduces hypochlorous acid (HOCl) production via decreasing NO production and inhibitory effect on chemotaxis and myeloperoxidase (MPO) (Alarcon De La Lastra and Villegas 2005). In addition, resveratrol in whole blood is able to inhibit O29, which is produced by stimulated human neutrophils (Rotondo et al. 1998; Cavallaro et al. 2003). Resveratrol reduces the production and secretion of IL‐8 and GM‐CSF, which are stimulated by IL‐1β (Alarcon De La Lastra and Villegas 2005; Culpitt et al. 2003). Resveratrol prevents DNA damage and inhibits tumorigenesis by two mechanisms: as a mutagen, it stimulates phase II enzymes such as quinone reductase, which have detoxification properties by inhibiting COX enzymes and cytochrome P4501A1 (Dubuisson et al. 2002; Mahyar‐Roemer et al. 2002). Also, as an antioxidant, it inhibits free radicals and prevents DNA damage (Jang et al. 1997). Increasing the expression of CD95L, p53, and p21 by resveratrol causes its growth‐inhibitory effect (Clément et al. 1998; Hsieh and Wu 1999; Hsieh et al. 1999). Therefore, differentiation and apoptosis are increased by resveratrol (Jang et al. 1997; Joe et al. 2002; Mahyar‐Roemer et al. 2001; Mahyar‐Roemer and Roemer 2001). As a result, resveratrol can inhibit all stages of carcinogenesis through different mechanisms (Alarcon De La Lastra and Villegas 2005). In general, resveratrol suppresses the activity of immune cells, modifies the synthesis of eicosanoids, and reduces the synthesis and secretion of pro‐inflammatory mediators. Moreover, it reduces the activity of enzymes such as COX‐1 or COX‐2, which are responsible for inflammatory mediators. Consequently, these mechanisms carried out the inhibitory effect of resveratrol on transcription factors like nuclear factor κB (NF‐κB) or activator protein‐1 (AP‐1) (Das and Das 2007a, 2007b).

### 3.2 Application of Resveratrol in Cancer

**FIGURE 3:**
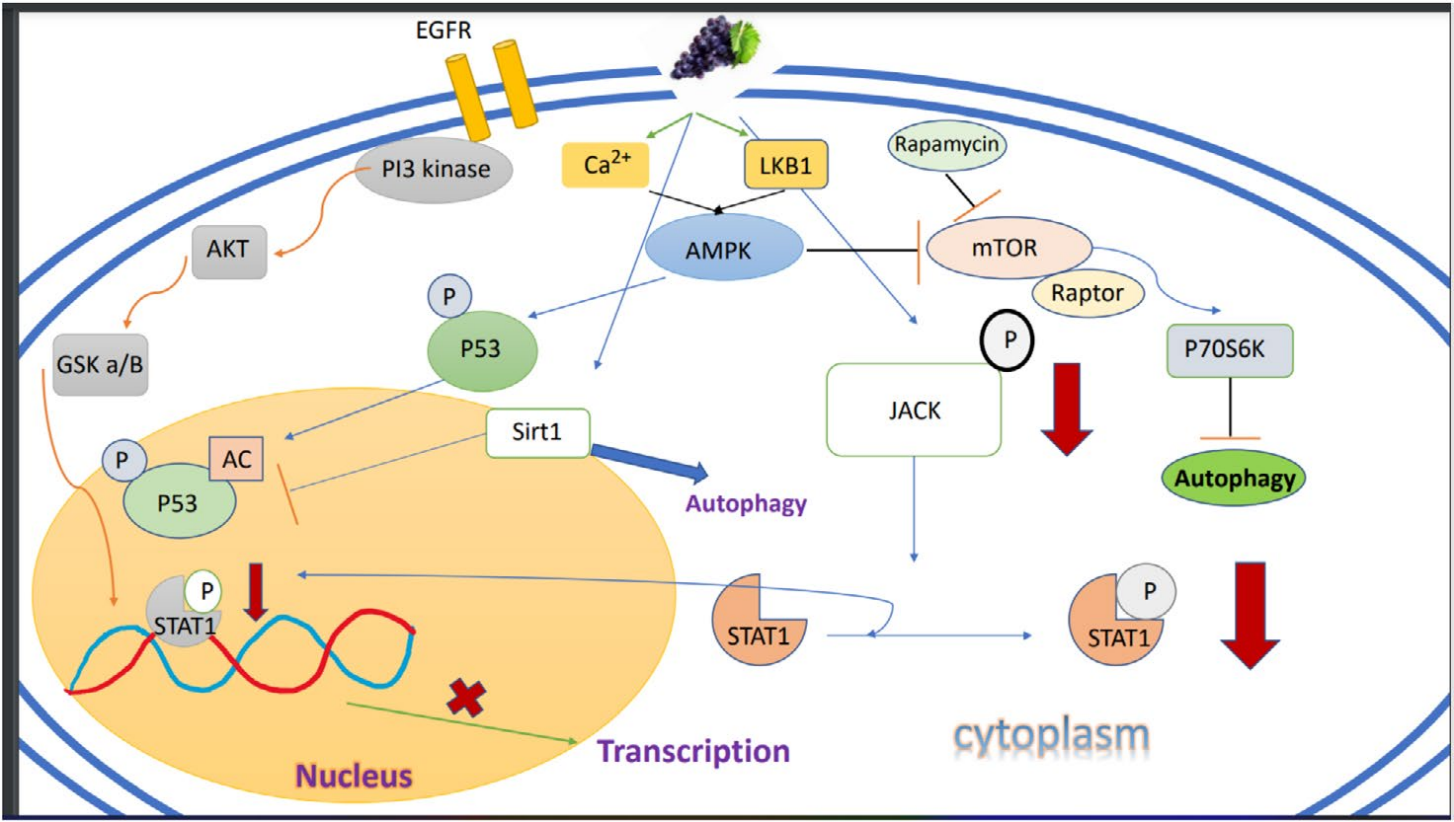
Resveratrol has an indirect effect on Ca^+2^ and LKB1, which inhibits mTOR with a direct effect on AMPK, which ultimately inhibits autophagy. Rapamycin also inhibits mTOR. Probably, through the path that EGFR has an effect on PI3 kinase, it eventually reduces the phosphorylation of STAT and prevents transcription. Resveratrol had a direct effect on JACK and can reduce phosphorylated STAT. Finally, it can reduce transcription. EGFR, epidermal growth factor receptor.

Cancer cells overexpress growth factors and their receptors and activate signaling pathways, including the phosphoinositide‐3‐kinase (PI3K)/Akt/mammalian target of rapamycin (mTOR), which can decrease apoptosis and accelerate cell proliferation Figure 3 (Martini et al. 2014). On the other hand, the activation of growth factor receptors increases proliferation due to the increased activity of the Ras‐mitogen‐activated protein kinase (Ras‐MAPK) cascade (Naidoo and Drilon 2016). Also, in many types of cancers, p53, which is a tumor suppressor that is dysregulated in cancer cells if not silenced (Barron et al. 2014). The results of various studies show that resveratrol inhibits EGFR, mTOR, NF‐kB, Akt, and JAK/STAT in lung cancer cells (Barron et al. 2014; Fan et al. 2015; Zhu et al. 2015; Ebi et al. 2009; Jung et al. 2013; Yu et al. 2013; Liu et al. 2011; Ulasli et al. 2013). In addition, resveratrol can be recognized as an activator of p53 and caspase tumor suppressor, which increases apoptosis (Barron et al. 2014; Zhu et al. 2015; Jung et al. 2013; Whyte et al. 2007; Yuan et al. 2015). Other roles of resveratrol include the activation of SIRT1, a histone deacetylase (Kulkarni and Cantó 2015; Chung et al. 2010), and increasing the activity of A549 lung cancer cells (Sun et al. 2013). In another study, it has been proven that resveratrol, with its effect on SIRT1, can inhibit NF‐κB activity (Buhrmann et al. 2015, 2016). This mechanism may be due to the decrease in phosphorylation and degradation of IκBα (Bhat and Pezzuto 2002). At the cellular level, resveratrol increases apoptosis and delays the cell cycle in the G1 → S transition phase (Bhat and Pezzuto 2002). Resveratrol prevents epithelial‐mesenchymal transition (EMT)‐related tumor invasion by regulating EMT markers, E‐cadherin and vimentin, and inhibiting Hedgehog (Hh) signaling (Li et al. 2014). Resveratrol can induce the differentiation of human promyelocytic cells (HL‐60 line) (Jang et al. 1997). Moreover, it reduces tumor growth via cell cycle arrest at G2/M phase in a mouse model (Carbó et al. 1999; Park et al. 2001). Resveratrol exerts its pro‐apoptotic and anti‐proliferative effects primarily on tumor cells, as demonstrated in human breast cancer cell lines (MCF‐7 and T47D), where it acted as an estrogen receptor agonist and modulated cell growth through receptor‐dependent mechanisms (Athar et al. 2007; Gehm et al. 1997). These findings were based on [^3^H]‐estradiol binding competition, estrogen‐responsive reporter gene, and cell proliferation assays, while no comparable cytotoxic effects were observed in normal cells (Gehm et al. 1997). In ER‐positive breast cancer cells, resveratrol behaves as a pathway‐selective estrogen receptor ligand that can activate estrogen‐responsive transcription without eliciting a classical mitogenic response, thereby dissociating ERα‐dependent anti‐inflammatory signaling from unchecked cell proliferation (Nwachukwu et al. 2014). Consistent with this concept, mechanistic studies in breast cancer models indicate that resveratrol reduces proliferation and other malignant traits by targeting multiple growth‐promoting pathways, including the suppression of c‐Myc‐driven phosphoglycerate kinase‐1 (PGK1) expression, which impairs glycolytic flux and limits the bioenergetic support for tumor cell growth (Gao et al. 2025). Moreover, resveratrol can counteract pro‐tumorigenic estradiol signaling at the level of cytoskeletal dynamics and growth‐factor crosstalk, exerting antiestrogenic effects on motility and survival pathways that are tightly coupled to cancer cell proliferation (Azios and Dharmawardhane 2005). These ER‐dependent and ER‐independent actions converge on key regulators of cell‐cycle progression and survival, providing a coherent mechanistic basis for the pro‐apoptotic and anti‐proliferative effects of resveratrol in breast cancer cells described above (Behroozaghdam et al. 2022). In addition to the synergistic effects of resveratrol with chemotherapy medications, it can also reduce people's resistance to these types of drugs, which have beneficial effects on chemotherapy‐resistant cancer cells (Yousef et al. 2017).

The approaches to prevent death from cancer are radiotherapy, immunotherapy, chemotherapy, and surgery (Gong et al. 2021). Radiotherapy destroys cancer tumors by emitting ionizing rays (IR) (Komorowska et al. 2022). Also, unlike chemotherapy, it does not have harmful effects on healthy cells and only causes the death of cancer cells (Calvaruso et al. 2019). Resveratrol leads to an increase in the radio resistance of healthy cells (Komorowska et al. 2022). Resveratrol in normal tissues reduces the cytotoxicity of radiotherapy (Mortezaee et al. 2020). Also, resveratrol can increase sensitivity to radiotherapy through its effects on apoptosis, anti‐inflammatory pathways, and oxidants (Wu et al. 2023). A study showed that resveratrol, by its anti‐apoptotic effect, can increase superoxide dismutase 2 (SOD2) as well as stimulate the expression of p53 and prevent the destruction of healthy intestinal cells (Zhang et al. 2017). Consumption of resveratrol before and after radiotherapy leads to the prevention of hematopoietic damage by reducing the production of ROS, NOX4, and also inhibiting NF‐κB and cyclooxygenase‐2 (COX‐2) (Zhang et al. 2017, 2013). On the other hand, it can increase the sensitivity of cancer cells to radiotherapy (Komorowska et al. 2022). Increased expression of STAT (signal transducer and activator of transcription) transcription factors in head and neck squamous cell carcinoma (HNSCC) leads to excessive growth and survival of cancer cells (Komorowska et al. 2022). The level of tumor suppressor p53 is reduced by the phosphorylated form of STAT3 (Komorowska et al. 2022). These conditions increase the activity of anti‐apoptotic proteins (Komorowska et al. 2022). This mechanism, by stimulating vascular endothelial growth factor (VEGF), matrix metalloproteinases‐2 and matrix metalloproteinases‐9 (MMP‐2 and MMP‐9), and inhibitors of apoptosis protein‐1 (IAP‐1), increases tumor resistance to chemotherapy agents and ionizing radiation (Mortezaee et al. 2020; Yu and Jove 2004). Resveratrol, by increasing the activity of suppressor of cytokine signaling 1 (SOCS‐1), a STAT3 inhibitor, leads to a decrease in the level of STAT3 phosphorylation in FaDu cells (Komorowska et al. 2022; Baek et al. 2016; Cotino‐Nájera et al. 2023). Due to this mechanism and the ability of resveratrol to reduce the response of cells to DNA damage, inhibit the cell cycle, and increase autophagy, resveratrol in the amount of 100 μM reduces cell proliferation, increases apoptosis, and increases cell sensitivity to radiation dose of 10 Gy (Baek et al. 2016; Mikami et al. 2019; Luo, Wang, et al. 2013). In a study conducted on prostate cancer cells, using X‐ray radiation in doses of 2–6 Gy and resveratrol with an amount of 25 or 500 μg/mL can increase apoptosis and delay the speed of cell responses to DNA damage, decrease cell proliferation, and suppress the cell cycle (Komorowska et al. 2022; Chen et al. 2017). Also, the combined therapeutic effects of resveratrol and radiotherapy include: decreasing the expression of cyclin B, cyclin D, and cell division protein kinase 2 (cdk2), and increasing the expression of anti‐proliferative molecules p15, p21, and p53 (Komorowska et al. 2022; Fang, DeMarco, and Nicholl 2012). Resveratrol combined with ionizing radiation up‐regulates the expression of perforin, but alone, up‐regulates the expression of granzyme B in PC‐3 and DU145 prostate cancer cells, which ultimately leads to increased apoptosis (Komorowska et al. 2022; Fang, Herrick, and Nicholl 2012). Another study confirms the ability of resveratrol to increase the sensitivity of cancer cells to ionizing radiation (Rashid et al. 2011). In such a way that the concentration of resveratrol in the amount of 2.5 and 5 μM increases the toxicity of PC cells in 2 Gy radiation therapy without affecting normal epithelial cells (Rashid et al. 2011). Also, in this study, it has been proven that radiotherapy in combination with resveratrol increases the activation of AMPK kinase and also inhibits Akt kinase in order to reduce gene expression involved in cell proliferation and increase cytotoxicity caused by radiation (Rashid et al. 2011; Tripathi et al. 2024). Inhibition of cell survival of radiation‐resistant PC cells, DU145 cells, due to the accumulation of ceramide, an important pre‐apoptotic signal, caused by ionizing radiation. It mediates by the synergistic effect of radiation and resveratrol (Scarlatti et al. 2007). Ceramide increases the activity of serine–threonine protein phosphatases, enzymes that reverse the actions of protein kinases by cleaving phosphate from serine and threonine residues in proteins (Hannun 1996). In addition, ceramide regulates protein phosphorylation, stress‐activated protein kinases, interleukin converting enzyme (ICE)‐like proteases, and the retinoblastoma gene product (Hannun 1996). A concentration of 50 μM resveratrol combined with a radiation dose of 5 Gy led to a decrease in the survival of melanoma cells (SW1 and WM35), the most aggressive type of skin cancer (Johnson et al. 2008). An animal study has been conducted on B16F10 mouse melanoma and CT26 mouse colon cancer cells, which showed that resveratrol can increase apoptotic cell death and loss of mitochondrial membrane potential by increasing ROS production. It can increase the sensitivity of cancer cells to radiation (Tak et al. 2012). In an in vitro study on non‐small cell lung cancer (NSCLC) A549 cells, resveratrol at a concentration of 20 μM, combined with radiotherapy doses ranging from 0 to 8 Gy, synergistically increased reactive oxygen species (ROS) production, DNA double‐strand breaks, and apoptosis‐independent molecular pathways, ultimately enhancing radio‐sensitization (Luo, Yang, et al. 2013). Also, resveratrol, by inhibiting the ability of A549 lung, prostate, colorectal, and melanoma cancer cells to regulate the intracellular calcium concentration through the calcium entry mechanism (SOCE), can reduce the effects of cellular radiation (Lopez et al. 2019; Collins et al. 2013; Lele et al. 2021). This stimulation effect occurs by expressing the stromal matrix interaction molecule (STIM1) and a calcium channel protein activated by calcium release (Orai1) (Lopez et al. 2019; Collins et al. 2013; Lele et al. 2021). Another mechanism that causes disease progression in cancer cells is the expression of SIRT1, which causes cell proliferation, angiogenesis, and resistance of cancer cells to treatment (Bosch‐Presegué and Vaquero 2011). However, this process may not be the same in different types of neoplastic cells (Bosch‐Presegué and Vaquero 2011). A study on lung cancer cells A549 and H460 showed resveratrol stimulates SIRT1, which regulates the amount of cyclin D1, leading to the inhibition of tumor cell proliferation and survival (Ji et al. 2018; Yang et al. 2013). Finally, another study in MCF‐7 cells showed that resveratrol, along with radiotherapy, reduces the activity of antioxidant enzymes and leads to the accumulation of ROS (Amini et al. 2021). It also increases the expression of Bax, p53, and caspase 8 and induces apoptosis (Komorowska et al. 2021).

Recent studies have further elucidated the radiosensitizing potential of resveratrol in various cancers. A recent study reviewed molecular mechanisms by which resveratrol enhances radiosensitivity through inhibition of NF‐κB and STAT3 pathways, increasing apoptosis in radioresistant cells such as those in hepatocellular and head and neck carcinomas (Cotino‐Nájera et al. 2023). Emerging evidence from another study demonstrated that resveratrol, combined with capsaicin, radiosensitizes colorectal tumors with efficacy comparable to 5‐fluorouracil but with lower hematological toxicity, suggesting a safer adjunct to radiotherapy (Amintas et al. 2025). An updated study highlighted resveratrol's role in combining ionizing radiation with natural radiosensitizers to overcome tumor resistance in lung and prostate cancer models (Tripathi et al. 2024). These findings, supported by recent analyses of resveratrol's anticancer mechanisms (Ren et al. 2025; Arif et al. 2024), underscore its potential but emphasize the need for clinical validation to address bioavailability challenges (Panigrahy et al. 2025). Our proposed framework incorporates these insights to optimize dosing and efficacy in future trials.

## 4 Resveratrol and Specific Radiotherapy Modalities

External beam photon radiotherapy with megavoltage X‐rays or γ‐rays remains the backbone of radiation treatment for the majority of solid tumors, typically delivered with conventional fraction sizes of 1.8–2.0 Gy or with moderately hypofractionated schedules (Koka et al. 2022).

In this setting, tumor cell elimination is largely mediated by the induction of DNA double‐strand breaks through both direct ionization and indirect effects via ROS, followed by activation of complex DNA damage response pathways and programmed cell‐death (Koka et al. 2022).

Plant‐derived polyphenols, including stilbenes and flavonoids, have been shown to modulate several of these key processes, such as ROS generation, antioxidant defenses, ataxia telangiectasia mutated and ataxia telangiectasia and Rad3 related (ATM/ATR) centered, DNA damage response (DDR) signaling, p53‐dependent checkpoints, and apoptosis (Carter et al. 2014).

On this basis, resveratrol is conceptually most suitable as an adjuvant to conventional and hypofractionated photon radiotherapy, where its ability to influence oxidative stress and DDR could enhance tumor radiosensitivity while sparing or protecting normal tissues (Carter et al. 2014; Khattar, Khan, Zaidi, Darvishikolour, et al. 2022).

Modern photon techniques such as three‐dimensional conformal radiotherapy, intensity‐modulated radiotherapy, and image‐guided radiotherapy mainly differ in geometric dose shaping and normal‐tissue sparing, but they share the same fundamental radiobiological mechanisms of DNA damage induction and repair as conventional external beam photon therapy (Jaffray 2012).

Consequently, the mechanistic actions described for resveratrol and related phytochemicals—namely modulation of ROS, reinforcement of endogenous antioxidant systems, interference with DDR signaling and regulation of pro‐ and anti‐apoptotic proteins—are expected to be relevant across these photon‐based modalities (Carter et al. 2014).

Particle therapies such as proton and carbon‐ion radiotherapy exhibit distinct physical dose distributions and, in the case of high‐LET ions, qualitatively different DNA damage patterns compared with photons, which can translate into altered normal‐tissue toxicity and therapeutic ratio (Chen et al. 2023).

Reviews of proton therapy highlight substantial dosimetric and clinical advantages in selected indications, but also note that the biological mechanisms still involve complex DNA damage, DDR activation and downstream cell‐death pathways similar to those triggered by photons (Chen et al. 2023).

Taken together, these considerations suggest that resveratrol might also influence responses to particle therapy through analogous mechanisms; however, because no specific experimental studies combining resveratrol with proton or heavy‐ion beams have been reported, its suitability in this context remains hypothetical and requires dedicated preclinical work before clinical translation can be proposed (Khattar, Khan, Zaidi, Darvishikolour, et al. 2022; Chen et al. 2023).

### 4.1 Limitations and Future Directions

Despite the promising preclinical effects of resveratrol in enhancing radio‐sensitization and inhibiting cancer cell proliferation, clinical applications face significant challenges. Reported side effects, such as gastrointestinal discomfort (e.g., nausea, diarrhea) and potential drug interactions at high doses, raise concerns about its safety profile (Shaito et al. 2020; Brockmueller et al. 2024; Brown et al. 2024). Furthermore, inconsistent clinical outcomes, possibly due to variations in dosing, patient populations, or study designs, limit its therapeutic reliability (Smoliga et al. 2013; Brown et al. 2024). One major limitation is the scarcity of clinical research on the efficacy and safety of resveratrol, particularly regarding its side effects during radiotherapy, as most available studies have been conducted on cell lines rather than in clinical settings, making it difficult to draw comprehensive clinical conclusions. These limitations underscore the need for well‐designed clinical trials to evaluate both efficacy and safety comprehensively. Future studies should focus on standardized dosing, thorough safety evaluation, and clinically relevant models to better assess resveratrol's efficacy and risks in radiotherapy.

## 5 Clinical Evidence

Several early‐phase clinical studies have evaluated resveratrol in patients with cancer or premalignant conditions, mainly focusing on safety, pharmacokinetics and exploratory biomarker endpoints rather than hard clinical outcomes (Carter et al. 2014; Khattar, Khan, Zaidi, Darvishikolour, et al. 2022; Walle 2011; Boocock et al. 2007).

Phase I trials in healthy volunteers and oncology populations have shown that oral resveratrol is generally well tolerated at single doses up to several grams per day, but plasma concentrations remain in the low micromolar or submicromolar range and exhibit substantial interindividual variability (Walle 2011; Boocock et al. 2007).

Beyond oncology, multiple randomized and non‐randomized trials in patients with type 2 diabetes, obesity or cardiovascular risk factors report modest and often inconsistent improvements in metabolic and inflammatory biomarkers, highlighting both the potential and the limitations of current dosing regimens and formulations (Öztürk et al. 2017; Szkudelski and Szkudelska 2011; Novelle et al. 2015).

Importantly, despite extensive preclinical evidence for radiosensitizing and radioprotective effects, no dedicated clinical trial has yet tested resveratrol prospectively as an adjuvant to radiotherapy, and available human data therefore provide only indirect support for such an application (Carter et al. 2014; Khattar, Khan, Zaidi, Darvishikolour, et al. 2022).

## 6 Translational Relevance and Future Directions

Although a large body of preclinical work demonstrates that resveratrol can modulate oxidative stress, DNA damage responses and cell death pathways in tumor and normal cells, the translational path towards routine clinical use in radiotherapy remains challenging (Carter et al. 2014).

Pharmacokinetic studies consistently show that orally administered resveratrol has low systemic bioavailability due to extensive first‐pass metabolism, so that the micromolar concentrations used in many in vitro radiosensitization experiments exceed those achievable with tolerable doses in humans (Walle et al. 2004).

Early‐phase clinical trials in oncology and other indications indicate that resveratrol is generally safe at daily doses up to several grams, but they also reveal variable target engagement and inconsistent biomarker responses, underscoring the need for optimized formulations and dosing schedules (Boocock et al. 2007; Novelle et al. 2015).

From a radiobiological perspective, the dual capacity of resveratrol to protect normal tissues while sensitizing tumor cells raises both opportunities and risks, making careful attention to tumor type, radiation modality, fractionation, timing of administration and potential drug–drug interactions essential in future trial designs (Khattar, Khan, Zaidi, Darvishikolour, et al. 2022; Kulkarni and Cantó 2015).

At present, therefore, resveratrol should be regarded as a promising but still investigational adjunct to radiotherapy, requiring well‐designed translational studies that integrate pharmacokinetic–pharmacodynamic modeling, rational patient selection and robust clinical endpoints before any claim of being “ready for prime time” can be justified (Carter et al. 2014).

## 7 Conclusion

Resveratrol, a polyphenol, shows potential anti‐cancer effects in preclinical studies, enhancing radio‐sensitization in cell lines via apoptosis, ROS production, and NF‐κB/STAT3 inhibition. It may also reduce serum glucose and protect normal tissues from radiotherapy damage. However, inconsistent clinical outcomes highlight the need for standardized trials to validate efficacy and safety. Overall, the available evidence indicates that resveratrol remains a promising yet investigational adjunct to radiotherapy. Addressing current inconsistencies will require carefully designed studies that integrate mechanistic insight with clinically meaningful endpoints, appropriate dosing strategies, and translational considerations.

## Author Contributions

S.E.K., A.Z., and A.R.D. contributed to the conception, design, and drafting of the manuscript. M.F., A.S., A.N., M.F., and A.H. contributed to manuscript drafting. All authors approved the final version for submission.

## Funding

This work was supported by the School of Nutritional Sciences and Dietetics, Tehran University of Medical Sciences, Tehran.

## Conflicts of Interest

The authors declare no conflicts of interest.

## Data Availability

The authors have nothing to report.
